# Association of health-related private transfers with treatment compliance of musculoskeletal disorders in the rural elderly: evidence from an underdeveloped region of China

**DOI:** 10.1186/s12891-020-03760-x

**Published:** 2020-11-14

**Authors:** Chaoyang Yan, Aichun Li, Qin Xiang, Jing Wang

**Affiliations:** 1 Department of Health Management, School of Medicine and Health Management, Tongji Medical College, Huazhong University of Science and Technology, Wuhan, 430030 Hubei China; 2 The Key Research Institute of Humanities and Social Science of Hubei Province, Huazhong University of Science and Technology, Wuhan, 430030 Hubei China; 3 Institute for Poverty Reduction and Development, Huazhong University of Science and Technology, Wuhan, 430030 Hubei China

**Keywords:** Health-related private transfers, Treatment compliance of musculoskeletal disorders, Poverty, Underdeveloped region of China

## Abstract

**Background:**

The prevalence and economic burdens of musculoskeletal disorders (MSD) are serious in rural China. In addition to formal support, health-related private transfers (HRPTs), including adult children transfers (ACTs), as well as relatives and friends transfers (RFTs), are very common in rural China. We explored the relationship between HRPTs and MSD treatment compliance and the heterogeneity of this relationship in terms of family socioeconomic status.

**Methods:**

A questionnaire survey was carried out in Enshi, Hubei, China by stratified random sampling in July 15–25,2018. A total of 2679 questionnaires on the economic burden of chronic diseases were collected. We deleted two questionnaires with missing data. The data was described using the mean and proportion. The Chi-square test and one-way ANOVA was used to compare each independent variable in the three groups, and ordered probit regression was used to analyse the relationship between each factor and treatment compliance. The heterogeneity of the effect was examined by group regression analysis of the samples with or without poverty.

**Results:**

In total, 853 samples with MSD were included in the analysis. The age was 70.27 ± 6.97 (mean +/− sd) years old, and the ADL was 11.64 ± 0.12, with more respondents being female (56.15%), partnered (73.51%), primary school educated (45.96%), working (57.91%), feeling poor in health (55.69%), lived alone or with a spouse (54.75%). Respondents with both ACTs and RFTs had better treatment compliance, and this was significant only in poor populations (*p* < 0.05). Under the same HRPTs’ condition, respondents who more compliant with MSD treatment were female (*p* < 0.01), had primary school education (*p* < 0.05), has self-reported poor (*p* < 0.01) and very poor (*p* < 0.05) health, had a high level of physical disability (*p* < 0.01), and were living with their children and grandchildren (*p* < 0.05). Respondents with more chronic diseases had poorer treatment compliance (*p* < 0.05).

**Conclusions:**

Only those in poverty who both have ACTs and RFTs are likely to have better treatment compliance for MSD. Promoting a culture of filial piety and fostering harmonious interpersonal relationships, policies that focus on groups that lack social support, and general formal support are essential for sustained access to treatment for MSD.

**Supplementary Information:**

The online version contains supplementary material available at 10.1186/s12891-020-03760-x.

## Background

Musculoskeletal disorders (MSD) are a series of chronic musculoskeletal system diseases, including the common rheumatoid arthritis (RA), lumbar disc herniation, osteoarthritis, gout, low back pain, neck pain, and other muscle and bone injuries. The prevalence of MSD is extremely common worldwide and it is estimated that the number of people suffering from MSD reached 1.31 billion in 2017, which is nearly one-fifth of the world’s population [1]. In rural China, the prevalence of MSD is about 40.3%, which is higher than that of urban areas (34.3%), ranking second only to circulatory diseases (156.8%) [2]. According to the fifth national health service survey of China in 2013, the prevalence of RA among the elderly in rural areas ranked fifth among chronic diseases, which is also higher than that of the elderly in urban areas [3].

MSD affect patients’ quality of life and cause great direct and indirect economic burden. The Global Burden of Disease Study 2017 estimates that the age-standardised disability-adjusted life year rate of MSD was as high as 1720.5 (per 100,000 population) [4]. In China, the years with disability for MSD reached 1774, ranking first among the leading causes of disability [5]. The average cost of hospitalisation for lumbar disc herniation was 9233.3 yuan in 2018 [2]. That year, the adjusted consumer price index (CPI) poverty line was 3535 yuan. A study on the economic burden of RA patients in China pointed out that the indirect financial burden of RA patients was $492.88 (± $1739.74) per patient per year, and the intangible costs for patients was $20,396.30 (± $31,145.10) [6].

Because MSD need frequent rehabilitation care and medication [7], factors linked with treatment compliance are worthy of examination. Adherence has been defined as: “the extent to which a person’s behaviour … corresponds with agreed recommendations from a healthcare provider” [8]. According to Kolt and colleagues, treatment compliance for MSD may encompass treatment attendance, concordance to physician’s advice, and undertaking of prescribed exercises [9]. Formal support, which has been shown to have a strong relationship with treatment compliance [10, 11]. Patients with RA who participated in patient support programs (help with medical expenses, nursing support, etc.) had a significant 14% reduction in drug discontinuation compared to the control group [12]. Compliance with biologic therapies for RA significantly decreased with increased weekly out-of-pocket payments and a higher proportion of therapy costs paid by patients [13]. However, patients with MSD are mostly elderly and have mobility difficulties [14], thus, general social support is likely necessary to promote MSD treatment compliance.

Health-related private transfers (HRPTs) as a means of social support, including not only from adult children (ACTs), but also from friends and relatives (RFTs), may affect treatment compliance of MSD [15, 16]. This may be due to several reasons. For one, private transfers still offer important financial support for the elderly in rural regions, especially in China [17]. Even if the social medical insurance achieves universal population coverage and rural residents are universally covered by social medical insurance, from the original New Cooperative Medical Scheme to the Urban-Rural Resident Basic Medical Insurance, the effective reimbursement rate is only 60% [18]. For another reason, family support is very important for the rural elderly, especially because of certain aspects of traditional Chinese culture, which emphasises filial piety and complex interpersonal networks [19, 20]. Therefore, it is possible for ACTs and RFTs to reduce the financial burden of MSD and improve treatment compliance to a certain extent.

China’s current poverty line is based on the constant price of 2300 yuan per capita net income in 2011. Those below this standard are poor households, otherwise they are non-poor. This study uses this standard to define poverty. Enshi Tujia and Miao Autonomous Prefecture is located in the remote mountainous area, which is one of the 14 concentrated poverty-stricken areas in China. Therefore, the selected survey areas are representative for China. Our study also has important implications to the less developed regions in the world.

However, with rapid urbanisation and the changing of family structure, private transfers are also changing in rural families [21]. Thus, the relationship between HRPTs and MSD treatment compliance in rural China deserves investigation. The purpose of this study is: (1) to analyse the relationship between the four types of HRPTs (ACTs only, RFTs only, both, and neither) and MSD treatment compliance, (2) to check the heterogeneity of family socioeconomic conditions in the relationship between private transfer and treatment compliance, and (3) to find other relevant factors affecting MSD treatment compliance under the same HRPTs’ conditions.

The study explains the relationship between HRPTs in poor areas and treatment compliance for MSD, which is meaningful for poor areas in China and the world. Because informal support is common not only in rural China, but also in other parts of the world. Studying its effect on musculoskeletal disease treatment compliance has implications for understanding the degree of informal support in poor rural areas and formulating more reasonable and effective medical insurance reimbursement policies.

## Methods

### Sampling process and data collection

From July 15 to 25, 2018, With the assistance of local village doctors, we conducted a questionnaire survey in the rural areas of the Enshi Prefecture, an underdeveloped region of Hubei Province, China, using multi-stage stratified random sampling. Sampling selection was conducted in the surveyed areas in a proportion of one third of the selected towns and villages. Two of Enshi’s six counties were chosen at random. Each county has an average of 15 townships and 5 townships are randomly selected. Each township has an average of nine villages, of which three are chosen. A total of 30 villages were selected. According to the matching design of 1:2, 10 poor households and 20 non-poor households were selected from each village.

We interviewed 913 elderly families and collected 2679 questionnaires (including elderly and family members). The subjects of the survey are elderly farmers who are engaged in agricultural work in the past and now. We found that 948 individuals suffered from MSD. Considering the age at which children become financially independent, we limited the age of respondents to over 55 years old, which eliminated 93 samples. Two samples had missing values regarding poverty, leaving 853 samples for analysis.

Our questionnaire has a standard questionnaire interpretation, and the study design does not involve specialized clinical knowledge (such as diagnosis and treatment regimen). A total of 15 investigators were recruited into our survey. we also provided special training for investigators, focusing on the uneducated population and conducting a pre survey. The investigation has also set up a special quality inspection team to oversee the investigation process and data entry. All respondents were given verbal informed consent, and we conducted an investigation on the premise that they were willing to answer our questions accurately. Therefore, we ensure that the respondents can accurately express their demographic information, physical feelings, and health service utilization.

### Variables selection and definitions

Table 1 shows the definition and assignment of variables. In this study, we used the self-reported four-classification scale to assess MSD treatment compliance (full compliance, most compliance, little compliance, or no compliance). Because there are only seven samples in the third category, we combined the third and fourth categories into one. HRPTs involved four types to compare the impact of transfer payments from different sources (none, ACTs only, RFTs only, or both).

**Table 1:** Definitions and coding of variables

Variables	Codes	Definitions
MSD Treatment compliance	1 = Full compliance; 2 = Most compliance; 3 = Little compliance or not at all	The degree of self-reported MSD treatment compliance
Age	Continuous variable	
Gender	1 = male; 2 = female	
Marital status	1 = single; 2 = partnered	
Poverty	1 = Yes; 2 = No	Whether the respondent was targeted for poverty alleviation
Job	1 = Yes; 2 = No	Whether the interviewee had a job during the survey
Duration	Continuous variable	How many years the respondent had MSD by the time of the survey
Ability of daily life activities (ADL)	Continuous variable	Whether there was difficulty in ten physical activities, 1 = no difficulty, 4 = total difficulty, the variable range is 10–40
HRPTs	1 = None; 2 = ACTs_only; 3 = RFTs_only; 4 = Both	The status of receiving transfer payments from children and relatives
Education	1 = Illiteracy; 2 = Primary school; 3 = Junior middle school and above	
Self-rated health (SRH)	1 = Good; 2 = general; 3 = poor; 4 = very poor	
Living arrangements	1 = Living alone or living with spouse; 2 = Living with grandchildren 3 = Living with children and grandchildren	Living arrangements with children and grandchildren
Comorbidities	1 = One type; 2 = two types; 3 = three types; 4 = Four types or more	How many types of chronic conditions did the respondent suffer from (including MSD)

Demographic characteristics are confounding variables that affect compliance with musculoskeletal treatment [22]. Thus, we incorporated gender, age, marital status, education, and work status as covariates. In addition, considering that family socioeconomic conditions directly affect their ability to pay for treatment, we also included family socioeconomic status into covariates. There is a study that pointed out that family-based exercise can prevent MSD [23], but in rural China, farmers rarely participate in exercise [24]. In addition, as far as we know, no related exercise programs aimed at preventing MSD were implemented in the investigation areas. Therefore, the relevant variables are not included. Comorbidity have been identified in previous study as being associated with treatment compliance for MSD, and was therefore included in our study [25]. Since the ability of daily living (ADL) of the elderly may affect their ability to receive treatment in medical institutions, and the self-rated health (SRH) status also partly reflects the psychological state of the elderly to a certain extent. So these two variables are incorporated into the model. In rural areas, phenomena such as empty nesters and left-behind children are common, and different living arrangements may receive different levels of support (including treatment costs and emotions), thus affecting treatment compliance. Therefore, the impact of the living arrangements has also been controlled in this study. There are three types of medical insurance in China: new rural cooperative medical system, basic medical insurance for urban employees and basic medical insurance for urban residents. The type of medical insurance in rural areas is the new rural cooperative medical system. In 2011, China has achieved near-universal health insurance coverage with more than 95% of the Chinese population covered by health insurance [26]. And the new rural cooperative medical insurance implement as the municipal-level. Therefore, there is no difference in reimbursement rate in the survey area. Hence, we regarded sample individuals as almost having no difference in health insurance, resulting in the exclusion of this variable from our model.

The questionnaire design and index selection were carried out for several rounds of expert consultation, taking into account the main characteristics of patients and ensuring the content validity.

Village doctors who are familiar with the local environment and the health status of local villagers are invited to do the communication and coordination of the survey and to assist the respondents in explaining the purpose of the survey. Rural physicians also corrected the respondent’s apparent recall bias.

### Statistical methods

Continuous variables are reported as means ± standard deviations. Categorical variables are presented as frequencies (%). One-way ANOVA was used to compare continuous variables, while the Chi-square test was used to compare categorical variables between groups. Ordered probit regression was applied to measure the effects of HRPTs and other potential confounding variables on MSD treatment compliance. The heterogeneity of the effects of HRPTs on treatment compliance was examined by grouping ordered probit regression according to poverty status.

We used the STATA software 13 to calculate the results. Statistical significance was set at *p*-value < 0.05.

### Ethical approval

The Ethics Committee of Tongji Medical College, Huazhong University of Science and Technology approved the research proposal (IRB: IORG0003571). All respondents provided informed consent before they were interviewed.

## Results

The four states of health-related transfer payments did not differ significantly between the three categories of treatment compliance for MSD. Table 2 shows the characteristics of all selected samples and their descriptive statistics results when the sample was grouped according to MSD treatment compliance. The results showed that the average age of the selected samples was 70.27 ± 6.97 years, the average duration of MSD was 11.56 ± 10.24 years, and the average ADL was 11.64 ± 0.12. A greater proportion of respondents were: female (56.15%), partnered (73.51%), primary school educated (45.96%), had a job (57.91%), felt poor in health (55.69%), lived alone or with his wife (54.75%), had no HRPTs (39.39%), and not in poverty (50.41%). Gender, activities of daily living (ADL), comorbidities, marital status, education, and poverty differed significantly between full compliance, most compliance, and little or no compliance groups.

**Table 2:** Characteristics of selected samples and results of univariate analysis

Variables	Overall	Treatment compliance for MSD			*P*
		Full	Most	Little or No	
Age (Mean/SD)	70.27 (6.97)	70.11 (7.02)	70.23 (6.87)	70.75 (7.19)	0.666
Gender(n/%)					0.001
Male	374 (43.85)	199 (62.78)	220 (55.28)	60 (43.48)	
Female	479 (56.15)	118 (37.22)	178 (44.72)	78 (56.52)	
Duration (Mean/SD)	11.56 (10.24)	11.15 (9.02)	11.48 (10.23)	12.76 (12.64)	0.299
ADL (Mean/SD)	11.64 (0.12)	12.23 (0.23)	11.31 (0.17)	11.22 (0.27)	< 0.01
Comorbidities(n/%)					0.014
One type	147 (17.23)	67 (21.14)	65 (16.33)	67 (21.14)	
Two types	246 (28.84)	90 (28.39)	122 (30.65)	90 (28.39)	
Three types	256 (30.01)	78 (24.61)	124 (31.16)	78 (24.61)	
Four types or more	204 (23.92)	82 (25.87)	87 (21.86)	82 (25.87)	
Marital status(n/%)					0.025
Single	226 (26.49)	97 (42.92)	88 (38.94)	41 (18.14)	
Partnered	627 (73.51)	220 (35.09)	310 (49.44)	97 (15.47)	
Education(n/%)					0.029
Illiteracy	306 (35.87)	112 (35.33)	151 (37.94)	43 (31.16)	
Primary school	392 (45.96)	158 (49.84)	176 (44.22)	58 (42.03)	
Junior middle school and above	155 (18.17)	47 (14.83)	71 (17.84)	31 (26.81)	
Job(n/%)					0.64
Yes	494 (57.91)	187 (58.99)	232 (58.29)	75 (54.35)	
No	359 (42.09)	130 (41.01)	166 (41.71)	63 (45.65)	
SRH (n/%)					0.197
Good	61 (7.15)	13 (21.31)	34 (55.74)	14 (22.95)	
General	170 (19.93)	63 (37.06)	76 (44.71)	31 (18.24)	
Poor	475 (55.69)	184 (38.74)	221 (46.53)	70 (14.74)	
Very poor	147 (17.23)	57 (38.78)	67 (45.58)	23 (15.65)	
Poverty(n/%)					0.001
Yes	423 (49.59)	183 (43.26)	179 (42.32)	61 (14.42)	
No	430 (50.41)	134 (31.16)	219 (50.93)	77 (17.91)	
Living arrangements(n/%)					0.264
Living alone or living with wife	467 (54.75)	159 (50.16)	226 (56.78)	82 (59.42)	
Living with grandchildren	125 (14.65)	50 (15.77)	59 (14.82)	16 (11.59)	
Living with children and grandchildren	261 (30.6)	108 (34.07)	113 (28.39)	40 (28.99)	
HRPTs(n/%)					0.282
None	336 (39.39)	117 (36.91)	152 (38.19)	67 (48.55)	
ACTs_only	247 (28.96)	91 (28.71)	122 (30.65)	34 (24.64)	
RFTs_only	117 (13.72)	44 (13.88)	57 (14.32)	16 (11.59)	
Both	153 (17.94)	65 (20.5)	67 (16.83)	21 (15.22)	

Age, Duration, Comorbidity, and ADL used one-way ANOVA, while the other variables used the Chi-square test

Only the elderly with both ACTs and RFTs showed better treatment compliance than those without HRPTs. In addition, we found that the effect was only significant among the poor. Table 3 shows the ordered probit regression results of the effects of each independent variable on MSD treatment compliance. In model 1, which included all selected samples, females and those with primary education had better treatment compliance than males and those who were illiterate, respectively. Elderly in poverty were more compliant than those who were not and the lower the SRH status, the better the treatment compliance. However, when a disability was involved, treatment compliance was improved when the handicap was greater. On the contrary, those with more chronic diseases had poorer treatment compliance. Elderly who lived with their children and grandchildren had better treatment compliance than those who lived alone or with their wives, and elderly with two types of transfer payment had better treatment compliance than those without any private transfers. In model 2, which only included the poverty sample, respondents with both ACTs and RFTs had better treatment compliance than those without any transfers. Model 3 shows the results of the non-poor sample. The effects of HRPTs on treatment compliance with MSD was not significant.

**Table 3:** Ordered probit results of HRPTs on the MSD treatment compliance

	Model1	Model2	Model3
Age	0.006 (0.007)	0.008 (0.010)	0.000 (0.010)
Gender (Ref: Male)	−0.296***(0.087)	− 0.271**(0.128)	− 0.390***(0.124)
Marital status (Ref: Single)	0.057 (0.098)	0.218 (0.134)	− 0.177 (0.149)
Poverty (Ref: Yes)	0.238***(0.080)		
Job (Ref: Yes)	0.077 (0.090)	−0.028 (0.127)	0.150 (0.132)
Duration	0.006 (0.004)	0.012**(0.006)	−0.000 (0.006)
ADL	−0.034***(0.012)	−0.040**(0.016)	− 0.031*(0.018)
HRPTs (Ref: None)			
ACTs_only	−0.117 (0.097)	−0.163 (0.140)	− 0.093 (0.138)
RFTs_only	−0.092 (0.124)	0.000 (0.176)	−0.190 (0.179)
Both	−0.240**(0.114)	−0.407**(0.170)	− 0.072 (0.157)
Education (Ref: Illiteracy)			
Primary school	−0.206**(0.094)	−0.291**(0.137)	− 0.132 (0.137)
Junior middle school and above	0.097 (0.125)	0.191 (0.191)	0.060 (0.176)
SRH (Ref: Good)			
General	−0.329*(0.171)	0.001 (0.287)	−0.624***(0.219)
Poor	−0.436***(0.160)	−0.413 (0.268)	− 0.482**(0.205)
Very poor	−0.425**(0.183)	− 0.281 (0.290)	−0.554**(0.250)
Living arrangements (Ref: Living alone or living with wife)			
Living with grandchildren	−0.177 (0.118)	−0.247 (0.171)	− 0.167 (0.172)
Living with children and grandchildren	−0.194**(0.092)	− 0.177 (0.128)	−0.232*(0.136)
Comorbidity (Ref: One type)			
Two types	0.276**(0.122)	0.347*(0.194)	0.203 (0.161)
Three types	0.547***(0.124)	0.440**(0.189)	0.632***(0.170)
Four types or more	0.343**(0.135)	0.360*(0.205)	0.336*(0.187)
/cut1	−0.446 (0.619)	−0.374 (0.901)	−1.937**(0.875)
/cut2	0.950 (0.619)	0.952 (0.901)	−0.432 (0.871)
Obs.	853	423	430
Pseudo R^2^	0.047	0.056	0.052

Standard errors are in brackets

Model 1 contains all selected samples, model 2 contains only poor samples, and model 3 contains only non-poor samples

*** *p* < 0.01, ** *p* < 0.05, * *p* < 0.1

## Discussion

Through the analysis of the effects of HRPTs and other variables on the MSD treatment compliance, we found that only the elderly with both ACTs and RFTs had better treatment compliance than those with no private transfers. Furthermore, we found that this effect was only significant among the poor. In addition, we also found that gender, education, SRH, ADL, comorbidities, and living arrangements also significantly affected MSD treatment compliance.

Elderly in poverty with both ACTs and RFTs had better treatment compliance, which may be related to the degree of social support and the status of work. Social support has been pointed out in a review to be related to treatment compliance with MSD. Text message-based social support intervention has been proved to be related to the treatment compliance of MSD [27, 28]. Our study suggested that HRPTs were associated with MSD. Respondents had two types of private transfers, which indicated that they benefited substantially from the culture of filial piety on one hand and that they had relatively rich interpersonal relationships and social networks on the other [29, 30]. Private transfers, from both inside and outside the family, enabled them to have more financial, emotional, and therapeutic support.

People in rural areas have higher labour participation with poorer individuals eager to obtain more income. Thus, poor people, even with MSD, tend to comply with treatment to maintain their work efficiency. One study points out that in rural China, older people with fewer family assets are more involved in labour [31]. Moreover, rural labour mostly involves manual labour. Therefore, in order to retain work, poor people will likely have better treatment compliance. Elderly from non-poor families face less economic pressure and rarely engage in long-term manual labour and HRPTs are more likely to occur during specific festivals, as it shows a form of courtesy and sympathy, but less likely to occur when paying for treatment. Thus, HRPTs may be used to treat other life-threatening diseases or may be used for leisure and entertainment. That was also why those with a higher level of disability maintained better treatment compliance.

In the case of the same transfer payment, treatment compliance was better in females. This may be due to the possibility that women are less tolerant of pain than men [32]. Respondents with primary education had better treatment compliance than those who were illiterate. This is likely because individuals who are literate have better health literacy, which is consistent with relevant studies [25]. Respondents with poorer perceived health status were more likely to continue treatment, thus, respondents with poorer SRH were more likely to comply. Respondents who lived with their children and grandchildren were more compliant than those who lived alone or with their wives. This may be due to the increase in medication and ongoing treatment reminders, as well as emotional support.

However, under the same transfer conditions, it seems unusual for the elderly with more chronic diseases to have poorer treatment compliance. A previous study have also shown that the comorbidity is associated with a higher likelihood of non-adherence to MSD, which is consistent with our findings [25]. One reason that may account for our research result is that, compared to other diseases, MSD is not one that requires urgent treatment. People with comorbidities generally require multiple medications, which could have undesirable side effects, such as impacts on their mood, which may make them feel uncomfortable and frustrated. This can make them less inclined to seek treatment for other chronic conditions, such as MSD, as they may find it hard to tolerate any more medication or long-term physical therapy [7]. Because MSD are not regarded as life-threatening [2], those with more than two types of chronic diseases tend to pay less attention to MSD treatment.

Our study revealed the relationship between HRPTs other than formal support (health insurance, welfare policies, etc.) and treatment compliance for MSD. Only those in poverty who had two kinds of private transfers were likely to have better treatment compliance. Therefore, it is meaningful to promote filial piety culture and promote harmonious interpersonal relationship. Formal policy benefits and financial transfers should also focus on groups with low informal transfers (such as those with fewer social networks and lack of filial cultural support). These all have implications for promoting equitable access to health care for MSD. However, with the development of urbanization, the interpersonal network in rural areas tends to weaken [21]. This suggests that although responders with two types of private transfers may have better treatment compliance for MSD, such HRPTs may not replace formal support completely. General welfare policies in poverty area are also needed.

### Limitations

This study is characterised by several limitations. The first is that the study did not subdivide MSD and only studied a specific pattern. This is because the respondent’s MSD are all self-reported, and there is no relevant medical record data to provide. Although all the residents included in our study have been told by doctors that they have musculoskeletal diseases, our survey objects are residents and lack of professional clinical knowledge, so it is difficult to accurately report the specific categories of MSD. Therefore, we included patients who were able to accurately report their own categories and patients who reported their own symptoms. Currently, there are two common approaches – using administrative data to calculate the Medication possession ratio (MPR) [33–35] and using questionnaires and scales, such as the 5-item Medication Adherence Compliance Report Scale (MARS-5) or the 4- or 8-item Morisky Medication Adherence Compliance Scale (MMAS) [36–38]. Due to the lack of medical records and the need for multiple treatments for MSD, these two methods of calculating treatment compliance are not applicable.

Secondly, our research did not focus on the effect of the changes in the costs required for MSD treatment compliance. Our research only focused on the association of four HRPTs types on MSD treatment compliance.

Lastly, our study is a cross-sectional study that illustrates the relationship between HRPTs and treatment compliance for MSD. Any related dynamic effects require further study of longitudinal data.

## Conclusions

It is important to promote a culture of filial piety and promote harmonious interpersonal relationships. Formal policy benefits and financial transfers should focus on groups with fewer informal transfers. These are conducive to the promotion of continuous treatment of musculoskeletal diseases. Health-related private transfers may not completely replace formal support. General welfare policies in poor areas are also necessary.

## Supplementary Information

**Additional file 1.** Health poverty risk identification questionnaire for rural elderly.

## Data Availability

The datasets used and/or analysed during the current study are available from the corresponding author on reasonable request.

## Abbreviations

MSD: Musculoskeletal disorders
HRPTs: Health-related private transfers
ACTs: Adult children transfers
RFTs: Relatives and friends transfers
RA: Rheumatoid arthritis
ADL: Ability of daily life activities
SRH: Self-rated health
