# Supplementary material for: Association of health-related private transfers with treatment compliance of musculoskeletal disorders in the rural elderly: evidence from an underdeveloped region of China
Source: BMC Musculoskelet Disord. 2020 Nov 14;21:747. doi: 10.1186/s12891-020-03760-x (PMC7666464; doi:10.1186/s12891-020-03760-x)
Supplement: Supplementary file 1 — Additional file 1. Health poverty risk identification questionnaire for rural elderly. [file 12891_2020_3760_MOESM1_ESM.docx]

**Health poverty risk identification questionnaire for rural elderly**

Questionnaire number：

Family address: ____township (town) ____village,___county, ___province

Hello! I am an investigator of Huazhong University of science and technology. I hope to know the general situation of your family, the health status of your family members, the utilization and guarantee of health services, and the family economy and other conditions, so as to provide the basis for identifying the health and poverty risks of the rural elderly and designing corresponding governance strategies. The questionnaire of this survey is only for statistical analysis. We will keep your and your family's information confidential. Participation in this study was entirely voluntary. We would like to thank you for your agreement to accept this survey. Thank you very much! The survey will take about 20-30 minutes.

| Overall family situation | | | | | | | | | | | | | |
| --- | --- | --- | --- | --- | --- | --- | --- | --- | --- | --- | --- | --- | --- |
|  | | Are families targeted at poverty alleviation? : (1) yes; (2) no | | | | | | | | |  | | |
|  | | Is the family a “Dibao” household ? (1) yes; (2) no | | | | | | | | |  | | |
|  | | The time to get to the nearest medical institution by the most convenient means of transportation: (1) within 10 minutes; (2) 10 to 20 minutes; (3) 20 to 30 minutes; (4) more than 30 minutes | | | | | | | | |  | | |
|  | | Number of permanent household residents in 2018 | | | | | | | | |  | | |
| Individual situation of family members | | | | | | | | | | | | | |
| （I） Basic information | | | | | | | 01 main respondents | 02 | 03 | 04 | | 05 | 06 |
| Who answers the following survey questions (as judged by the investigator) (1) Answer in person (2) by others | | | | | | |  |  |  |  | |  |  |
|  | Gender: (1) male; (2) female | | | | | |  |  |  |  | |  |  |
|  | Age (as at 1 July 2018, in years) | | | | | |  |  |  |  | |  |  |
|  | The relationship between the member and the main interviewees: (1) main interviewees (2) spouse; (3) children or son-in-law or daughter-in-law; (4) parents or parents in law or parents in law; (5) grandparents; (6) grandchildren; (7) brothers / sisters; (8) others | | | | | |  |  |  |  | |  |  |
|  | Marital status: (1) unmarried; (2) married; (3) widowed; (4) divorced | | | | | |  |  |  |  | |  |  |
|  | Nature of household registration: (1) agriculture; (2) non agriculture | | | | | |  |  |  |  | |  |  |
|  | Education level: (1) illiterate; (2) primary school; (3) junior high school; (4) senior high school or technical secondary school; (5) junior college; (6) undergraduate; (7) graduate student or above | | | | | |  |  |  |  | |  |  |
|  | （Inquiry for persons over 16 years old) Main types of work (single choice): (1) farming; (2) self-employed; (3) stable employment; (4) flexible employment; (5) unemployed; (6) Retired personnel; (7) Students at school (8) Others (please specify) _______ | | | | | |  |  |  |  | |  |  |
|  | Reasons for unemployment: (1) Unemployment; (2) Retirement due to age; (3) Inability to work due to physical reasons; (4) Resignation/lay-off/closure; (5) Others | | | | | |  |  |  |  | |  |  |
| （II） Physical health | | | | | | | 01 | 02 | 03 | 04 | | 05 | 06 |
|  | Height (unit: cm) | | | | | |  |  |  |  | |  |  |
|  | Weight (unit: kg) | | | | | |  |  |  |  | |  |  |
|  | Self-perceived health status: (1) very good; (2) relatively good; (3) fair; (4) relatively bad; (5) very bad | | | | | |  |  |  |  | |  |  |
|  | (Only for subjects aged 60 and above) Whether there is hearing impairment: (1) Yes; (2) No | | | | | |  |  |  |  | |  |  |
|  | Smoking frequency: (1) never smoke; (2) occasionally smoke (within 3 days a month); (3) smoke frequently (3 days a week and less); (4) smoke frequently (more than 3 days a week); (5) Quit smoking | | | | | |  |  |  |  | |  |  |
|  | Drinking frequency: (1) Never drink alcohol (skip to 25); (2) Drink occasionally (within 3 days a month); (3) Drink frequently (3 days a week or less); (4) Drink frequently (every week) 3 days or more); (5) Already abstained from alcohol (skip to 25) | | | | | |  |  |  |  | |  |  |
|  | Drinking type: (1) Drinking spirits such as white wine/foreign wine; (2) Low-alcohol alcohol such as rice wine/red wine/beer/rice wine | | | | | |  |  |  |  | |  |  |
|  | (Required for each person) Do you suffer from chronic diseases (judging criteria for six months or more diagnosed by the doctor): (1) Yes; (2) No (skip to 32) | | | | | |  |  |  |  | |  |  |
|  | Disease code: (fill in the diagnosis code in descending order of severity of illness, if there is no code, please write the diagnosis directly) | | | | | A |  |  |  |  | |  |  |
|  |  |  |  |  |  | B |  |  |  |  | |  |  |
|  |  |  |  |  |  | C |  |  |  |  | |  |  |
|  |  |  |  |  |  | D |  |  |  |  | |  |  |
|  |  |  |  |  |  | E |  |  |  |  | |  |  |
|  | Where is the diagnosis of each disease (corresponding to the above question, and single choice): (1) Village clinic; (2) Township health center; (3) County-level medical institution; (4) City-level medical institution; ( 5) Provincial and above medical institutions; (6) Private clinics | | | | | A |  |  |  |  | |  |  |
|  |  |  |  |  |  | B |  |  |  |  | |  |  |
|  |  |  |  |  |  | C |  |  |  |  | |  |  |
|  |  |  |  |  |  | D |  |  |  |  | |  |  |
|  |  |  |  |  |  | E |  |  |  |  | |  |  |
|  | Duration of each disease/symptom (unit: year, accurate to 0.5) | | | | | A |  |  |  |  | |  |  |
|  |  |  |  |  |  | B |  |  |  |  | |  |  |
|  |  |  |  |  |  | C |  |  |  |  | |  |  |
|  |  |  |  |  |  | D |  |  |  |  | |  |  |
|  |  |  |  |  |  | E |  |  |  |  | |  |  |
|  | Where is the main treatment for each disease (respectively correspond to the above question, and single choice): (1) Village clinic; (2) Township health center; (3) County-level medical institution; (4) City Level medical institutions; (5) Provincial level and above medical institutions; (6) Private clinics; (7) At home/not treated | | | | | A |  |  |  |  | |  |  |
|  |  |  |  |  |  | B |  |  |  |  | |  |  |
|  |  |  |  |  |  | C |  |  |  |  | |  |  |
|  |  |  |  |  |  | D |  |  |  |  | |  |  |
|  |  |  |  |  |  | E |  |  |  |  | |  |  |
|  | Compliance with treatment for each disease (respectively correspond to the above questions, and single-choice): (1) Full compliance; (2) Most compliance; (3) Little compliance; (4) ) not at all | | | | | A |  |  |  |  | |  |  |
|  |  |  |  |  |  | B |  |  |  |  | |  |  |
|  |  |  |  |  |  | C |  |  |  |  | |  |  |
|  |  |  |  |  |  | D |  |  |  |  | |  |  |
|  |  |  |  |  |  | E |  |  |  |  | |  |  |
|  | The changes of each disease from the past (respectively correspond to the above question, and single choice): 1) worsening; (2) maintaining; (3) improving; (4) curing | | | | | A |  |  |  |  | |  |  |
|  |  |  |  |  |  | B |  |  |  |  | |  |  |
|  |  |  |  |  |  | C |  |  |  |  | |  |  |
|  |  |  |  |  |  | D |  |  |  |  | |  |  |
|  |  |  |  |  |  | E |  |  |  |  | |  |  |
|  | (Required for each person) Whether it is disabled (based on the disability certificate): (1) Yes; (2) No (skip to 35) | | | | | |  |  |  |  | |  |  |
|  | Disability level: (1) first level; (2) second level; (3) third level; (4) fourth level | | | | | |  |  |  |  | |  |  |
|  | Specific types of disability: (1) physical disability; (2) brain damage/mental disability; (3) blind or semi-blind; (4) deaf or semi-deaf; (5) dumb or severe stuttering | | | | | |  |  |  |  | |  |  |
|  | (Required for everyone except healthy infants and young children) Put on clothes: (1) No difficulty; (2) Difficulty but still able to complete (only partial help is needed); (3) Difficulty and need great help; (4) Unable to complete, totally dependent on others | | | | | |  |  |  |  | |  |  |
|  | Bathing: (1) No difficulty; (2) Difficulty but still able to complete (only partial help is needed); (3) Difficulty and need great help; (4) Unable to complete, totally dependent on others | | | | | |  |  |  |  | |  |  |
|  | Eating: (1) No difficulty; (2) Difficulty but still able to complete (only partial help is needed); (3) Difficulty and need great help; (4) Unable to complete, totally dependent on others | | | | | |  |  |  |  | |  |  |
|  | Getting in and out of bed: (1) No difficulty; (2) Difficulty but still able to complete (only partial help is needed); (3) Difficulty and need great help; (4) Unable to complete, totally dependent on others | | | | | |  |  |  |  | |  |  |
|  | Go to the toilet: (1) No difficulty; (2) Difficulty but still able to complete (only partial help is needed); (3) Difficulty and need great help; (4) Unable to complete, totally dependent on others | | | | | |  |  |  |  | |  |  |
|  | Stool control: (1) No difficulty; (2) Difficulty but can still be completed (only partial help is needed); (3) Difficulty and need great help; (4) Unable to complete, totally dependent on others | | | | | |  |  |  |  | |  |  |
|  | Control urination: (1) No difficulty; (2) Difficulty but still able to complete (only partial help is needed); (3) Difficulty and need great help; (4) Unable to complete, totally dependent on others | | | | | |  |  |  |  | |  |  |
|  | Freshen up: (1) No difficulty; (2) Difficulty but still able to complete (only partial help is needed); (3) Difficulty and need great help; (4) Unable to complete, totally dependent on others | | | | | |  |  |  |  | |  |  |
|  | Walking on the ground: (1) No difficulty; (2) Difficulty but still able to complete (only partial help is needed); (3) Difficulty and need great help; (4) Unable to complete, totally dependent on others | | | | | |  |  |  |  | |  |  |
|  | Up and down stairs: (1) No difficulty; (2) Difficulty but still able to complete (only partial help is needed); (3) Difficulty and need great help; (4) Unable to complete, totally dependent on others | | | | | |  |  |  |  | |  |  |
|  | Duration of the above-mentioned earliest difficult situation so far (unit: year, accurate to 0.5): | | | | | |  |  |  |  | |  |  |
|  | (When there are difficulties in any of 31-40) The status of the member has changed from the past: (1) deterioration; (2) maintenance; (3) improvement; (4) normal function | | | | | |  |  |  |  | |  |  |
|  | (When there are difficulties in any of 31-40) Do you need help from others or totally rely on the help of others in daily life (excluding healthy infants and young children): (1) Yes; (2) No | | | | | |  |  |  |  | |  |  |
|  | At present, the most important ways to care for family members who have difficulties in daily life and need help are (single choice): (1) Home care; (2) Community care; (3) Institutional care (medical institution); (4) Institutional care (maintenance institution); (5) Unattended; (6) Others (please specify) _______ | | | | | |  |  |  |  | |  |  |
|  | In the past year, the expenses paid for hiring a caregiver (excluding members of the family) or entrusting a care institution to take care of the above-mentioned members (unit: yuan) | | | | | |  |  |  |  | |  |  |
| （III）Mental health status (asked only to the main respondent) | | | | | | | | | | | | | |
|  | (Required for the main interviewee) Are you anxious or depressed? (1) No anxiety or depression; (2) Moderate anxiety or depression; (3) Extreme anxiety or depression | | | | | |  | | | | | | |
|  | Worrying about some small things: (1) little or no (<1 day); (2) not too much (1-2 days); (3) sometimes or half the time (3-4 days); ( 4) Most of the time (5-7 days) | | | | | |  | | | | | | |
|  | It is difficult to concentrate when doing things: (1) little or no (<1 day); (2) not too much (1-2 days); (3) sometimes or half the time (3-4 days) ; (4) Most of the time (5-7 days) | | | | | |  | | | | | | |
|  | 1. Feeling depressed: (1) little or no (<1 day); (2) not too much (1-2 days); (3) sometimes or half the time (3-4 days); ( 4) Most of the time (5-7 days) | | | | | |  | | | | | | |
|  | I feel strenuous to do anything: (1) little or no (<1 day); (2) not too much (1-2 days); (3) sometimes or half the time (3-4 days) ; (4) Most of the time (5-7 days) | | | | | |  | | | | | | |
|  | Full of hope for the future: (1) little or no (<1 day); (2) not too much (1-2 days); (3) sometimes or half the time (3-4 days); (4) ) Most of the time (5-7 days) | | | | | |  | | | | | | |
|  | Feeling scared: (1) little or no (<1 day); (2) not too much (1-2 days); (3) sometimes or half the time (3-4 days); (4) large Most of the time (5-7 days) | | | | | |  | | | | | | |
|  | Poor sleep: (1) Little or no sleep (<1 day); (2) Not too much (1-2 days); (3) Sometimes or half the time (3-4 days); (4) Most of the time (5-7 days) | | | | | |  | | | | | | |
|  | Very pleasant: (1) little or no (<1 day); (2) not too much (1-2 days); (3) sometimes or half the time (3-4 days); (4) large Most of the time (5-7 days) | | | | | |  | | | | | | |
|  | Feeling lonely: (1) little or no (<1 day); (2) not too much (1-2 days); (3) sometimes or half the time (3-4 days); (4) large Most of the time (5-7 days) | | | | | |  | | | | | | |
|  | Feeling unable to continue living: (1) Little or no (<1 day); (2) Not too much (1-2 days); (3) Sometimes or half the time (3-4 days); (4) ) Most of the time (5-7 days) | | | | | |  | | | | | | |
| （IV）Medical service utilization | | | | | | | 01 | 02 | 03 | 04 | | 05 | 06 |
|  | (Required for each person) Has the disease been sick in the past four weeks: (1) Yes; (2) No | | | | | |  |  |  |  | |  |  |
|  | Have you seen an outpatient clinic in the past four weeks: (1) Yes; (2) No (skip to 67) | | | | | |  |  |  |  | |  |  |
|  | Number of outpatient visits within four weeks (unit: times) | | | | | |  |  |  |  | |  |  |
|  | The total cost of outpatient service in four weeks (unit: yuan) (please refer to the receipt as far as possible) | | | | | |  |  |  |  | |  |  |
|  | Including: Out-of-pocket expenses (excluding reimbursement and expenses in personal medical accounts) (unit: yuan) (as far as possible based on the receipt) | | | | | |  |  |  |  | |  |  |
|  | In addition to outpatient expenses, the total cost of travel, accommodation, food, escort and other expenses for outpatient visits within four weeks (unit: yuan) | | | | | |  |  |  |  | |  |  |
|  | Did you buy drugs by yourself in the past four weeks: (1) Yes; (2) No (skip to 70) | | | | | |  |  |  |  | |  |  |
|  | The total cost of self-purchasing drugs within four weeks (unit: yuan) (as far as possible, subject to the receipt) | | | | | |  |  |  |  | |  |  |
|  | Including: Out-of-pocket expenses (excluding reimbursement and expenses in personal medical accounts) (unit: yuan) (as far as possible based on the receipt) | | | | | |  |  |  |  | |  |  |
|  | In 2017, the number of hospitalizations diagnosed by a doctor (unit: times) (for example, answer 0 times, skip to 80) | | | | | |  |  |  |  | |  |  |
|  | In 2017, the actual number of hospitalizations (unit: times) | | | | | |  |  |  |  | |  |  |
|  | (if the number of hospital stay should be different from the actual number of hospitalization) the reasons for not being hospitalized: (1) economic reasons; (2) unnecessary; (3) no time; (4) ineffective measures; (5) other reasons | | | | | |  |  |  |  | |  |  |
|  | Number of hospitalization days (fill in from most to least, unit: day) | | | | the first time | |  |  |  |  | |  |  |
|  |  |  |  |  | the second time | |  |  |  |  | |  |  |
|  |  |  |  |  | the third time | |  |  |  |  | |  |  |
|  |  |  |  |  | the fourth time | |  |  |  |  | |  |  |
|  |  |  |  |  | Remaining cumulative | |  |  |  |  | |  |  |
|  | Diagnosis of diseases in previous hospitalizations: (fill in the diagnostic code one by one, if there is no code, please write the diagnosis directly) | | | | the first time | |  |  |  |  | |  |  |
|  |  |  |  |  | the second time | |  |  |  |  | |  |  |
|  |  |  |  |  | the third time | |  |  |  |  | |  |  |
|  |  |  |  |  | the fourth time | |  |  |  |  | |  |  |
|  |  |  |  |  | Remaining cumulative | |  |  |  |  | |  |  |
|  | Corresponding hospitalization institutions: (1) township health centers; (2) county-level medical institutions; (3) municipal-level medical institutions; (4) provincial-level and above medical institutions | | | | the first time | |  |  |  |  | |  |  |
|  |  |  |  |  | the second time | |  |  |  |  | |  |  |
|  |  |  |  |  | the third time | |  |  |  |  | |  |  |
|  |  |  |  |  | the fourth time | |  |  |  |  | |  |  |
|  |  |  |  |  | Remaining cumulative | |  |  |  |  | |  |  |
|  | Reasons for the selection of the corresponding inpatient institutions: (1) Self-service selection; (2) Doctor referral; (3) Other____ | | | | the first time | |  |  |  |  | |  |  |
|  |  |  |  |  | the second time | |  |  |  |  | |  |  |
|  |  |  |  |  | the third time | |  |  |  |  | |  |  |
|  |  |  |  |  | the fourth time | |  |  |  |  | |  |  |
|  |  |  |  |  | Remaining cumulative | |  |  |  |  | |  |  |
|  | The total cost of hospitalization in one year (unit: yuan) (as far as possible based on the receipt) | | | | | |  |  |  |  | |  |  |
|  | Including: Out-of-pocket expenses (excluding reimbursement and expenses in personal medical accounts) (unit: yuan) (as far as possible based on the receipt) | | | | | |  |  |  |  | |  |  |
|  | In addition to hospitalization expenses, the total cost of travel, accommodation, food, escort and other expenses for hospitalization in one year (unit: yuan) | | | | | |  |  |  |  | |  |  |
|  | (Inquiry by non-unemployed persons over 16 years of age) Whether there was any missed work in 2017: (1) Yes; (2) No | | | | | |  |  |  |  | |  |  |
|  | In 2017, the total lost time (unit: month, accurate to 0.5 month) | | | | | |  |  |  |  | |  |  |
|  | The reasons for the loss of work are as follows: (1) absence due to illness; (2) absence due to disability; (3) absence due to caring for others; (4) other reasons | | | | | |  |  |  |  | |  |  |
|  | Average monthly income before work in 2017 (unit: yuan) | | | | | |  |  |  |  | |  |  |
|  | In addition to the above hospital out of pocket expenses, other out of Pocket Medical and health expenses in 2017 (unit: yuan) (subject to the documents as far as possible) | | A. Vaccination | | | |  |  |  |  | |  |  |
|  |  |  | B. physical examination | | | |  |  |  |  | |  |  |
|  |  |  | C. Physical therapy of traditional Chinese and Western Medicine (such as acupuncture, cupping, traction, etc.) | | | |  |  |  |  | |  |  |
|  |  |  | D. Insurance premium of primary medical treatment insurance | | | |  |  |  |  | |  |  |
|  |  |  | E. Commercial or supplementary medical insurance premiums | | | |  |  |  |  | |  |  |
|  |  |  | F. Medical expenses | | | |  |  |  |  | |  |  |
|  |  |  | G. Cost of care | | | |  |  |  |  | |  |  |
|  |  |  | H. other | | | |  |  |  |  | |  |  |
| （V）Family Economic Status and Social Support | | | | | | | | | | | | | |
|  | Number of permanent residents in previous households (unit: person) | | Number of permanent residents in households in 2015 | | | | | | | | |  | |
|  |  |  | Number of permanent residents in households in 2016 | | | | | | | | |  | |
|  |  |  | Number of permanent residents in households in 2017 | | | | | | | | |  | |
|  | Annual family total income of the previous year (unit: yuan) | | 2015 | A. Wage income (including laborers, odd jobs, etc.) | | | | | | | |  | |
|  |  |  |  | B. Household operating income from agricultural production, raising livestock and poultry (first clarify the types of agricultural products and poultry and livestock, and then ask them one by one) | | | | | | | |  | |
|  |  |  |  | C. Income from foreign investment and property leasing (such as land transfer, etc.) | | | | | | | |  | |
|  |  |  |  | D. Compensatory income from national agricultural support policies (such as pensions, agricultural subsidies, subsistence allowances, subsidies for the disabled, subsidies for military personnel, subsidies for senior citizens, student grants, etc.) | | | | | | | |  | |
|  |  |  |  | E. Child support | | | | | | | |  | |
|  |  |  | 2016 | A. Wage income (including laborers, odd jobs, etc.) | | | | | | | |  | |
|  |  |  |  | B. Household operating income from agricultural production, raising livestock and poultry (first clarify the types of agricultural products and poultry and livestock, and then ask them one by one) | | | | | | | |  | |
|  |  |  |  | C. Income from foreign investment and property leasing (such as land transfer, etc.) | | | | | | | |  | |
|  |  |  |  | D. Compensatory income from national agricultural support policies (such as pensions, agricultural subsidies, subsistence allowances, subsidies for the disabled, subsidies for military personnel, subsidies for senior citizens, student grants, etc.) | | | | | | | |  | |
|  |  |  |  | E. Child support | | | | | | | |  | |
|  |  |  | 2017 | A. Wage income (including laborers, odd jobs, etc.) | | | | | | | |  | |
|  |  |  |  | B. Household operating income from agricultural production, raising livestock and poultry (first clarify the types of agricultural products and poultry and livestock, and then ask them one by one) | | | | | | | |  | |
|  |  |  |  | C. Income from foreign investment and property leasing (such as land transfer, etc.) | | | | | | | |  | |
|  |  |  |  | D. Compensatory income from national agricultural support policies (such as pensions, agricultural subsidies, subsistence allowances, subsidies for the disabled, subsidies for military personnel, subsidies for senior citizens, student grants, etc.) | | | | | | | |  | |
|  |  |  |  | E. Child support | | | | | | | |  | |
|  | Total annual Household Expenditure in 2017 (unit: yuan) | | A. Food expenditure (including rice, noodles, oil, vegetables, eggs, milk, etc., excluding tobacco and alcohol) | | | | | | | | |  | |
|  |  |  | B. Medical expenses (check with 84) | | | | | | | | |  | |
|  |  |  | C. Education expenditure | | | | | | | | |  | |
|  |  |  | D. Water, electricity and fuel costs | | | | | | | | |  | |
|  |  |  | E. Telephone, internet and other communication expenses | | | | | | | | |  | |
|  |  |  | F. Tobacco and alcohol charges | | | | | | | | |  | |
|  |  |  | G. Transportation | | | | | | | | |  | |
|  |  |  | H. Commodity expenditure | | | | | | | | |  | |
|  |  |  | I. Hiring personnel costs | | | | | | | | |  | |
|  |  |  | J. clothing | | | | | | | | |  | |
|  |  |  | K. tourism | | | | | | | | |  | |
|  |  |  | L. Furniture appliances | | | | | | | | |  | |
|  |  |  | M. Culture and entertainment | | | | | | | | |  | |
|  |  |  | N. Fund other people or relationships | | | | | | | | |  | |
|  |  |  | O. other expenses | | | | | | | | |  | |
|  | In 2017, borrowings or loans for health-related expenses of family members (unit: yuan) | | | | | | | | | | |  | |
|  | In 2017, how much help can I get from relatives and friends due to family members’ health problems: (1) Get great help; (2) Get some help; (3) Get no help at all; (4) Not clear or not happening | | | | | | | | | | |  | |
|  | In 2017, Whether to get health-related transfer payments from children?(1) yes; (2) no | | | | | | | | | | |  | |
|  | In 2017, Whether to get health-related transfer payments from relatives and friends? (1) yes; (2) no | | | | | | | | | | |  | |
|  | In 2017, due to health problems of family members, relatives and friends provided unpaid care services for a total of years (unit: month) | | | | | | | | | | |  | |
| This is the end of the questionnaire, thank you for your cooperation! | | | | | | | | | | | | | |
